# Effects of dietary xylanase supplementation on growth performance, intestinal health, and immune response of nursery pigs fed diets with reduced metabolizable energy

**DOI:** 10.1093/jas/skae026

**Published:** 2024-01-27

**Authors:** Jonathan T Baker, Marcos Elias Duarte, Sung Woo Kim

**Affiliations:** Department of Animal Science, North Carolina State University, Raleigh, NC 27695, USA; Department of Animal Science, North Carolina State University, Raleigh, NC 27695, USA; Department of Animal Science, North Carolina State University, Raleigh, NC 27695, USA

**Keywords:** intestinal health, metabolizable energy, mucosa-associated microbiota, nursery pig, xylanase

## Abstract

This study aimed to investigate the effects of xylanase on growth performance and intestinal health of nursery pigs fed diets with reduced metabolizable energy (**ME**). One hundred ninety-two pigs at 8.7 kg ± 0.7 body weight (**BW**) after 7 d of weaning were allotted in a randomized complete block design with initial BW and sex as blocks. Eight dietary treatments consisted of 5 ME levels (3,400, 3,375, 3,350, 3,325, and 3,300 kcal ME/kg) below the NRC (2012) requirement and 4 levels of xylanase (0, 1,200, 2,400, and 3,600 XU/kg) to a diet with 3,300 kcal ME/kg. All pigs received their respective treatments for 35 d in 2 phases, pre-starter (14 d) and starter (21 d). On day 35, eight pigs in 3,400 kcal/kg (CON), 3,300 kcal/kg (LE), and 3,300 kcal/kg + 3,600 XU xylanase/kg (LEX) were euthanized to collect jejunal tissues and digesta for the evaluation of mucosa-associated microbiota, intestinal immune response, oxidative stress status, intestinal morphology, crypt cell proliferation, and digesta viscosity as well as ileal digesta to measure apparent ileal digestibility. Data were analyzed using the MIXED procedure on SAS 9.4. The LE increased (*P* < 0.05) jejunal digesta viscosity, tended to have decreased (*P* = 0.053) relative abundance of *Prevotella*, and tended to increase (*P* = 0.055) *Lactobacillus.* The LE also increased (*P* < 0.05) the concentration of protein carbonyl whereas malondialdehyde, villus height (**VH**), villus height to crypt depth ratio (**VH:CD**), apparent ileal digestibility (**AID**) of nutrients, and finally average daily feed intake were decreased (*P* < 0.05). The LE did not affect average daily gain (**ADG**). The LEX decreased (*P* < 0.05) digesta viscosity, increased (*P* < 0.05) the relative abundance of *Prevotella*, decreased (*P* < 0.05) *Helicobacter*, decreased (*P* < 0.05) the concentration of protein carbonyl, tended to increase (*P* = 0.065) VH, and decreased (*P* < 0.05) VH:CD and crypt cell proliferation. Moreover, LEX increased (*P* < 0.05) the AID of dry matter and gross energy and tended to increase (*P* = 0.099; *P* = 0.076) AID of crude protein, and ether extract. The LEX did not affect ADG but did tend to decrease (*P* = 0.070) fecal score during the starter phase. Overall, reducing ME negatively affected intestinal health parameters and nutrient digestibility without affecting growth. Supplementation of xylanase mitigated some of the negative effects observed by ME reduction on intestinal health and digestibility of nutrients without affecting growth.

## Teaser

Reduction of metabolizable energy through the removal of dietary fat can have detrimental effects on immune status, intestinal health, and mucosa-associated microbiota of nursery pigs. The supplementation of an exogenous xylanase in energy-reduced feeds up to 100 kcal ME/kg can mediate some of the negative effects of reduced dietary fat inclusion in nursery pigs.

## Introduction

Highly digestible and nutrient-dense feedstuffs are commonly used in nursery pig feeds to combat the detrimental effects imposed by weaning stress in addition to compensating for the subsequent reduction of feed intake. Energy density in feeds is a critical component affecting feed intake, as an increase in energy level can decrease feed intake and a decrease in energy level can hamper growth and protein deposition (Southern et al., 1989). The requirement of metabolizable energy (**ME**) for pigs suggested by the NRC increased when the 11th revision was published (NRC, 1998, 2012). However, according to Tokach et al. (1995), varying ME levels in feeds did not affect the growth performance of newly weaned pigs. One practical method of increasing the energy in feeds is to include supplemental fats in the formulation. However, in the case of newly weaned pigs, their ability to digest typical supplemental fats is limited reaching only 65% to 80% whereas milk fat is highly digestible at 95% (Cera et al., 1988; Lauridsen, 2020). In addition, the growing demand for fats and oils by external industries such as biodiesel production (Toldrá-Reig et al., 2020), has increased the price and decreased the availability of typical supplemental fats that can be used in feeds (Mielke, 2018). Dietary fats go beyond just meeting the energy specification of the feed but become a source of fatty acids with specific functional roles in the body (Kim et al., 2007; Liu, 2015). Dietary supplementation of functional fatty acids including omega-3 series fatty acids, lysophospholipids, and conjugated linolenic acid has been shown to modulate the intestinal immune status, intestinal morphology, epithelial barrier function, and intestinal microbiota (Bassaganya-Riera et al., 2001; Jang et al., 2020; Lauridsen, 2020). In addition, increasing levels of dietary fat in feeds slow down the passage rate through the digestive tract, allowing relatively more time for increased digestion and absorption of other nutrients (Valaja and Siljander-Rasi, 2001). Poultry fat, the fat source used in this study, is composed of approximately 20% linoleic acid, a polyunsaturated fatty acid (**PUFA**) (NRC, 2012). Long-chain PUFA is a precursor of prostanoids, which can stimulate the recovery of intestinal barrier function (Ferrer and Moreno, 2010). In pigs, PUFA supplementation has been shown to improve the repair of previously damaged small intestines (Lopez-Pedrosa et al., 1998; López-Pedrosa et al., 1999; Jacobi et al., 2012).

Price volatility and availability of feedstuffs used in pig feeds drive the need to efficiently use readily available and economically favorable coproducts in formulation. Typically, increased inclusion of coproducts results in an increase in nonstarch polysaccharides (**NSP**) that are not digestible by endogenous enzymes of pigs and thus the supplementation of feed enzymes targeting specific types of NSP has been implemented (Kim and Baker, 2003; Adeola and Cowieson, 2011).

Common cereal grains and related coproducts contain variable amounts of NSP that can alter digesta viscosity in the small intestine and subsequently lead to morphological changes on the mucosal surface (Bedford and Classen, 1992; Lindberg, 2014), increased proliferation of potentially pathogenic microorganisms (Shakouri et al., 2009; Moita et al., 2022), and an increase or decrease in the retention time and passage rate of digesta depending on the type of NSP (Jørgensen et al., 1996; Wilfart et al., 2007; Schop et al., 2019; Chassé et al., 2023; Dorado-Montenegro et al., 2023). Xylanase targets the glycosidic bonds present in xylan, a structurally diverse family of NSP that share a β-1,4-linked xylopyranose backbone as a common feature (Scheller and Ulvskov, 2010). Arabinoxylan (AX) is one of the most common NSP present in cereal grains such as corn, wheat, barley, and their associated coproducts and is composed of a linear β-1,4 xylan backbone with α-1,3 and/or α-1,2-L-arabinofuranose branch points with varying degrees of substitution (Wang et al., 2020; Baker et al., 2021). Once ingested, AX can form viscous gels, increasing the viscosity of digesta and blocking the accessibility of endogenous digestive enzymes to feed particles entrapped in the viscous structure (McDonald et al., 2001; Passos et al., 2015; Duarte et al., 2019). Traditionally, xylanase has been used to increase the utilization of nutrients in the feed by lessening digesta viscosity as the xylanase breaks down the structural architecture of AX (Bedford and Schulze, 1998; Bedford, 2018). Past studies have indeed reported increases in nutrient digestibility with xylanase supplementation (Newman, 2014; Kiarie et al., 2016; Passos et al., 2023), in addition to increases in average daily gain (**ADG**) and feed conversion (Myers and Patience, 2014; Yang et al., 2016). Further research has elucidated functional roles of xylanase in pig feeds such as the ability of xylanase to beneficially modulate intestinal health by reducing the digesta viscosity (Passos et al., 2015; Tiwari et al., 2018), releasing prebiotic xylooligosaccharides and arabinoxylooligosaccharides (Masey-O’Neill et al., 2014; Dale et al., 2020), positively impacting the relative abundance and diversity of intestinal microbiota (Zhang et al., 2018; Chen et al., 2020; Moita et al., 2022), reducing inflammatory and oxidative damage products in the jejunum (Duarte et al., 2019; Petry et al., 2020), and thus improving intestinal integrity (Tiwari et al., 2018; Chen et al., 2020).

Based on previous findings, it is hypothesized that reducing ME by 100 kcal ME/kg feed through the reduction of supplemental fat during the nursery phase may negatively affect growth performance and intestinal health. It is also hypothesized that xylanase supplementation will mediate some of the negative effects on growth performance and intestinal health seen by the reduction of supplemental fat and thus reduced ME. To test this hypothesis, the objective of the study was to investigate the effects of xylanase supplementation on growth performance and intestinal health of nursery pigs fed diets with reduced ME.

## Materials and Methods

The procedure of this study was reviewed and approved by North Carolina State University Animal Care and Use Committee (Raleigh, NC). The experiment was conducted at the Central Crops Research Station (Clayton, NC).

### Experimental design, animals, and diets

One hundred and ninety-two newly weaned pigs (96 barrows and 96 gilts) at 7.3 ± 0.6 kg body weight (**BW**) were weaned at day 21 of age and fed a common early weaner diet for 7 d. After 7 d feeding, pigs reached 8.7 ± 0.7 kg BW and were allotted to eight dietary treatments in a randomized complete block design with initial BW and sex as blocks. Three pigs were housed per pen. The power test was completed to have 8 replicates per treatment, requiring 64 pens to handle eight dietary treatments. Dietary treatments consisted of diets with five levels of ME meeting or below NRC (2012) at 3,400 kcal ME/kg (CON), 3,375 kcal ME/kg, 3,350 kcal ME/kg, 3,325 kcal ME/kg, and 3,300 kcal ME/kg (LE) as well as diets with 4 levels of xylanase to LE (3,300 kcal ME/kg) at 0 XU/kg, 1,200 XU/kg, 2,400 XU/kg, and 3,600 XU/kg feed (LEX). All dietary treatments retained the same lysine to energy ratio. All pigs received their respective treatments for 35 d in two phases: pre-starter phase (14 d) and starter phase (21 d). Pigs had ad libitum access to water and feed during the entire 35 d period. Xylanase was supplied by CJ BIO (Seoul, Korea). All dietary treatments were offered in the form of mash feed. Titanium dioxide (0.4%) was mixed into the diet as an indigestible external marker and fed during the last 5 d of the starter phase.

Compositions of the experimental diets from all phases are shown in Table 1. All experimental diets were produced at Feed Mill Educational Unit (Raleigh, NC) at North Carolina State University. All experimental diets were sampled and sent to the North Carolina Department of Agriculture and Consumer Services for proximate analysis of nutrient composition.

**Table 1.** Composition of experimental diets with varying metabolizable energy (ME)

		ME^1^, kcal/kg									
Item	Early weaner	Pre-starter					Starter				
	Basal	3,400	3,375	3,350	3,325	3,300^2^	3,400	3,375	3,350	3,325	3,300^2^
Feedstuff, %											
Corn, yellow dent	23.6	27.5	28.0	28.5	29.0	29.5	32.7	33.2	33.7	34.2	34.7
Wheat	15.0	20.0	20.0	20.0	20.0	20.0	25.0	25.0	25.0	25.0	25.0
Whey permeate	24.0	17.0	17.0	17.0	17.0	17.0	10.0	10.0	10.0	10.0	10.0
Soybean meal	17.0	24.0	24.0	24.0	24.0	24.0	27.0	27.0	27.0	27.0	27.0
Poultry meal	10.0	4.0	4.0	4.0	4.0	4.0	—	—	—	—	—
Blood plasma	6.0	—	—	—	—	—	—	—	—	—	—
Fish meal	—	2.0	2.0	2.0	2.0	2.0	—	—	—	—	—
Poultry fat	2.0	3.0	2.5	2.0	1.5	1.0	2.5	2.0	1.5	1.0	0.50
L-Lys HCl	0.50	0.56	0.56	0.56	0.56	0.56	0.47	0.47	0.47	0.47	0.47
L-Met	0.22	0.20	0.20	0.20	0.20	0.20	0.14	0.14	0.14	0.14	0.14
L-Thr	0.15	0.18	0.18	0.18	0.18	0.18	0.14	0.14	0.14	0.14	0.14
L-Val	0.00	0.04	0.04	0.04	0.04	0.04	0.02	0.02	0.02	0.02	0.02
Dicalcium phosphate	0.10	0.40	0.40	0.40	0.40	0.40	0.65	0.65	0.65	0.65	0.65
Limestone, ground	0.80	0.75	0.75	0.75	0.75	0.75	1.00	1.00	1.00	1.00	1.00
Vitamin premix^3^	0.03	0.03	0.03	0.03	0.03	0.03	0.03	0.03	0.03	0.03	0.03
Mineral premix^4^	0.15	0.15	0.15	0.15	0.15	0.15	0.15	0.15	0.15	0.15	0.15
Salt	0.22	0.22	0.22	0.22	0.22	0.22	0.22	0.22	0.22	0.22	0.22
ZnO	0.25	—	—	—	—	—	—	—	—	—	—
BMD30	0.03	—	—	—	—	—	—	—	—	—	—
Calculated composition											
Dry matter, %	91.07	90.52	90.46	90.40	90.34	90.28	89.85	89.79	89.73	89.67	89.61
ME, kcal/kg	3,409	3,401	3,376	3,351	3,326	3,301	3,357	3,332	3,307	3,282	3,258
Crude protein, %	24.74	21.71	21.76	21.80	21.84	21.88	20.11	20.15	20.20	20.24	20.28
Crude fiber, %	—	—	—	—	—	—	1.72	1.73	1.74	1.75	1.76
SID^5^ Lys, %	1.50	1.35	1.35	1.35	1.36	1.36	1.23	1.23	1.23	1.23	1.23
SID Met + Cys, %	0.82	0.74	0.74	0.74	0.74	0.74	0.68	0.69	0.69	0.69	0.69
SID Thr, %	0.89	0.79	0.80	0.80	0.80	0.80	0.73	0.73	0.73	0.73	0.73
SID Trp, %	0.26	0.22	0.22	0.22	0.22	0.22	0.22	0.22	0.22	0.22	0.22
SID Val, %	1.01	0.86	0.86	0.86	0.87	0.87	0.78	0.79	0.79	0.79	0.79
SID Ile, %	0.81	0.75	0.75	0.75	0.75	0.75	0.71	0.71	0.71	0.71	0.71
Ca, %	0.86	0.80	0.80	0.80	0.80	0.80	0.71	0.71	0.71	0.71	0.71
STTD^6^ P, %	0.46	0.40	0.40	0.40	0.41	0.41	0.33	0.33	0.33	0.33	0.33
Total P, %	0.68	0.63	0.63	0.64	0.64	0.64	0.56	0.56	0.56	0.56	0.56
Analyzed composition											
Dry matter, %	91.24	89.98	89.95	89.84	89.80	89.73	88.23	88.73	88.62	88.44	88.40
Gross energy, kcal	4,175	4,120	4,131	4,108	4,086	4,019	4,069	4,040	4,022	4,009	3,921
Crude protein, %	23.37	21.60	20.12	20.18	20.41	19.80	18.74	18.77	18.93	18.82	19.03
Crude fiber, %	—	—	—	—	—	—	2.47	2.33	2.61	1.10	1.06
NDF, %	6.77	6.75	6.66	6.09	6.80	5.68	6.70	7.02	6.56	6.37	6.34
ADF, %	3.25	3.04	3.09	2.84	3.17	3.35	2.49	2.83	2.67	2.36	2.31
Ca, %	0.95	0.87	0.76	0.79	0.80	0.80	0.79	0.70	0.71	0.72	0.67
P, %	0.66	0.58	0.55	0.59	0.58	0.55	0.50	0.50	0.50	0.51	0.50

^1^Metabolizable energy level at 3,400 kcal/kg (CON), 3,375 kcal/kg, 3,350 kcal/kg, 3,325 kcal/kg, and 3,300 (LE) kcal/kg of feed.

^2^Xylanase supplemented at 0, 1,200, 2,400, and 3,600 XU/kg (LEX) to a diet with 3,300 kcal/kg (LE).

^3^The vitamin premix provided per kilogram of complete diet: 6,614 IU of vitamin A as vitamin A acetate, 992 IU of vitamin D3, 19.8 IU of vitamin E, 2.64 mg of vitamin K as menadione sodium bisulfate, 0.03 mg of vitamin B12, 4.63 mg of riboflavin, 18.52 mg of D-pantothenic acid as calcium panthonate, 24.96 mg of niacin, and 0.07 mg of biotin.

^4^The trace mineral premix provided per kilogram of complete diet: 33 mg of Mn as manganous oxide, 110 mg of Fe as ferrous sulfate, 110 mg of Zn as zinc sulfate, 16.5 mg of Cu as copper sulfate, 0.30 mg of I as ethylenediamine dihydroiodide, and 0.30 mg of Se as sodium selenite.

^5^Standardized ileal digestible.

^6^Standardized total tract digestible phosphorus.

### Experimental procedures and sample collection

The BW and feed intake were recorded every 7 d to calculate average BW, ADG, average daily feed intake (**ADFI**), and gain:feed (G:F). Fecal scores were recorded every 3 d based on a 1 to 5 scale: (1) very hard and dry feces, (2) firm stool, (3) normal stool, (4) loose stool, and (5) watery stool with no shape as described in Weaver and Kim (2014).

At the end of day 35 of feeding, a total of 24 pigs in three dietary treatments were selected for further research to investigate the intestinal health. Specifically, one pig representing a median BW of each pen in 3,400 kcal ME/kg (CON), 3,300 kcal ME/kg (LE), and 3,300 kcal ME/kg + 3,600 XU xylanase/kg (LEX) were selected and euthanized by a captive bolt gun followed by exsanguination and removal of the gastrointestinal tract for sample collection. Mid-jejunum segments (3 m after the pyloric duodenal junction) were taken digesta was collected in 50-mL Falcon tubes to measure digesta viscosity. Mucosal samples from the mid-jejunum were scraped by a glass slide and collected in Eppendorf tubes (2 mL), then put into liquid nitrogen immediately and stored at −80 °C for subsequent mucosa-associated microbiota measurements. Mid-jejunum tissues were rinsed with 0.9% saline solution and collected in a 50-mL Falcon tube with 10% buffered formaldehyde to evaluate histology. Ileal digesta was collected in a 100 mL container and put on the ice, then stored at −20 °C for measurement of apparent ileal digestibility (**AID**) of nutrients.

### Digesta Viscosity

Following the procedure by Passos et al. (2015) and Duarte et al. (2019), samples of jejunum digesta from 50 mL tubes were divided into 2 falcon tubes (15 mL) and centrifuged at 1,000 × *g* at 4 °C for 10 min to obtain the liquid phase. The liquid phase was then removed and transferred to an Eppendorf tube (2 mL) to centrifuge at 10,000 × *g* at 4 °C for 10 min. The supernatant obtained was transferred to another Eppendorf tube (1.5 mL) for further measurement. About 0.5 mL of digesta supernatant was placed in the viscometer (Brookfield Digital Viscometer, Model DV-II Version 2.0, Brookfield Engineering Laboratories Inc., Stoughton, MA), set at 25 °C. The viscosity measurement was the average between 45.0 s^−1^ and 22.5 s^−1^ shear rates, and the viscosity values were recorded as apparent viscosity in centipoise (**cP**).

### Relative abundance and diversity of jejunal mucosa-associated microbiota

Mid-jejunum mucosa samples were utilized for mucosa-associated microbiome sequencing using 16S rRNA gene sequence analysis. The DNA was extracted from the mucosa samples using the DNA Stool Mini Kit (#51604, Qiagen; Germantown, MD) as previously described by Duarte et al. (2020). Extracted DNA samples were then sent to the Genomics Department of Mako Medical Laboratories (Raleigh, NC) for 16S rRNA gene sequencing. In short, extracted DNA samples were prepared for the template on an Ion Chef and then sequenced on the Ion S5 system (Thermo Fisher Scientific Inc., Waltham, MA, USA). The variable regions analyzed were V2, V3, V4, V6, V7, V8, and V9 of the 16S rRNA gene and amplified via Ion 16S Metagenomics Kit (Thermo Fisher Scientific Inc.). Hypervariable regions were processed using Torrent Suite software (version 5.2.2) (Thermo Fisher Scientific Inc.) to produce.bam files for further analysis. The taxonomy was assigned against the GreenGenes (anybody) and MicroSeq (experts) databases, specific primers for microbiota. Alpha diversity rare fraction plot generation, and the Operational Taxonomic Unit (**OTU**) table generation were performed by the Ion Reporter Software Suite (version 5.2.2) of bioinformatics analysis tools (Thermo Fisher Scientific Inc.) with 98% similarity. The alpha diversity was calculated using Chao1 index (Chao, 1984), Shannon index (Shannon and Weaver, 1949), and Simpson index (Simpson, 1949). The Ion Reporter’s Metagenomics 16S workflow powered by Qiime (version w1.1) was used to analyze the samples. The depth of sequencing coverage was >1,000× sample preparation. To initiate the statistical analysis of the microbiota, OTU data were transformed to relative abundance as previously described by Kim et al. (2019). The OTU with a relative abundance <0.5% within each level were combined as “Others”.

### Inflammatory cytokines, immunoglobulins, and oxidative damage products

Jejunal mucosa samples were weighed (1 g) and suspended in 1 mL of phosphate-buffered saline (**PBS**) on ice, then homogenized using a tissue homogenizer (Tissuemiser; Thermo Fisher Scientific Inc.). Following Holanda and Kim (2021), the processed samples were then transferred into a new 2 mL microcentrifuge tube and centrifuged at 14,000 × *g* for 15 min. The supernatant was pipetted into five aliquots and stored at −80 °C.

The concentration of total protein, interleukin-8 (**IL-8**), tumor necrosis factor-alpha (TNF-α), immunoglobulin G (**IgG**), immunoglobulin A (**IgA**), protein carbonyl, and malondialdehyde (**MDA**) were measured by using commercial kits based on the instruction manual. The OD value was read by the ELISA plate reader (Synergy HT, BioTek Instruments, Winooski, VT) and software (Gen5 Data Analysis Software, BioTek Instruments). The corresponding concentrations were calculated according to the absorbance of the standard curves and instruction manual.

The homogenized mucosal supernatant was diluted (1:60) in PBS to get the appropriate range (20 to 2000 μg/mL), and then the total protein concentration was measured by using Pierce BCA Protein Assay Kit (#23225, Thermo Fisher Scientific Inc.) as described by Holanda et al. (2020). The absorbance was measured at 562 nm and the concentration of total protein were further used to normalize the concentration of other measurements in mucosa. The concentration of IL-8 was measured by using Porcine IL-8/CXCL8 DuoSet ELISA kit (#DY535, R&D Systems) as described by Jang and Kim (2019). All samples were diluted in reagent diluent to 1:5 to analyze. Absorbance was read at 450 nm and corrected at 570 nm. The TNF-α concentration was measured by using the following Porcine TNF-α Immunoassay Kit (#PTA00, R&D Systems, Minneapolis, MN, USA) as described by Cheng et al. (2021). Absorbance was read at 450 nm and corrected at 570 nm. The concentration of TNF-α was expressed as pg/mL protein. The concentration of IgA and IgG was measured by using the ELISA kits (E101-102 and E101-104, Bethyl Laboratories, Inc., Montgomery, TX) as described by Holanda et al., (2020). The mucosal supernatants were diluted with PBS to 1:1200 and 1:2400, respectively, to get the appropriate working range for measurement. Absorbance was read at 450 nm and the concentration was expressed as μg/mg of protein. The concentration was expressed as pg/mL protein. Protein carbonyl was measured by using OxiSelect Protein Carbonyl ELISA Kit (#STA-310, Cell Biolabs, Inc., San Diego, CA, USA) as described by Moita et al. (2021). All supernatants were diluted in PBS to get 10 µg/mL before measurement. The standard was prepared that range was from 0.375 to 7.5 nmol/mg protein. All processes are conducted following the manufacturer’s protocol. The absorbance was measured at 450 nm and the concentration was described as nmol/mg protein. The concentration of MDA in mucosa was measured by using OxiSelect TBARS MDA Quantitation Assay Kit (#STA-330, Cell Biolabs, Inc.) as described by Moita et al. (2021). The working range of the standard is from 0.98 to 125 µM/L. The absorbance was read under 532 nm wavelength. The concentration was calculated according to the standard and expressed as µmol/mg protein.

### Intestinal morphology and Ki-67 in crypt cells

Two sections of mid jejunum per pig were fixed in 10% formalin and then transferred to a 70% ethanol solution for 2 d. The processed samples were sent to North Carolina State University Histology Laboratory (College of Veterinary Medicine, Raleigh, NC) for dehydration, embedment, staining, and immunohistochemistry of Ki-67 proteins. Villus height (**VH**), villus width, and crypt depth (**CD**) were measured using a microscope Olympus CX31 (Lumenera Corporation, Ottawa, Canada) with software Infinity 2-2 digital CCD. In each slide, 10 intact villi and their associated crypts were measured as described by Cheng et al. (2021). The villus length was measured from the top of the villus to the junction of villus and crypt; the villus width was measured in the middle of the villus; the crypt depth was measured from the junction of villus and crypt to the bottom of the crypt. The villus height to crypt depth (**VH:CD**) ratio was calculated using the villus height divided by the CD. Images of 10 intact crypts from each slide were cropped, and the ImageJS software was used for calculating the percentage of Ki-67 positive cells to total cells in the crypt and to count the number of Ki-67 positive cells per crypt. All analyses of the intestinal morphology were executed by the same person. The averages of the 10 measurements per pig were calculated and reported as one number per pig.

### Apparent ileal digestibility

Titanium dioxide was added at an inclusion rate of 0.4% to starter diets to serve as an indigestible marker to determine the AID of nutrients. Ileal digesta were freeze-dried for 48 h (24D 48, Virtis, Gardiner, NY). The concentration of titanium dioxide in the feed and digesta were measured based on the approach of Myers et al. (2004). The feed and digesta samples were used to measure the content of dry matter (**DM**, method 934.01), crude fiber (method 978.10), and ether extract (**EE**, method 2003.06) were measured based on AOAC (2006). Gross energy (**GE**) was measured using a bomb calorimeter (Model 6200, Parr Instrument Company, Moline, IL). The nitrogen content was measured using TruSpec N Nitrogen Determinator (LECO CN-2000, LECO Corp., St. Joseph, MI) and the CP concentration was calculated (6.25 × N). The AID of DM, GE, EE, and CP were calculated by using the following function:


$$\begin{array}{l} AID=\left\{1\left[\left(\frac{TiO_{2feed}}{TiO_{2digesta}}\right)\times \;\left(\frac{Nutrient_{digesta}}{Nutrient_{feed}}\right)\right]\right\}\times 100 \end{array}$$

Which TiO_2feed_ and TiO_2digesta_ were the measured concentration of titanium dioxide in the feed and in the digesta; Nutrient_digesta_ and Nutrient_feed_ were the measured concentration of nutrient in the digesta and in the feed as previously described by Moita et al. (2021).

### Statistical analysis

Data were analyzed with MIXED procedure by using SAS 9.4 (SAS Inc., Cary, NC, USA). Main effect was dietary treatment, considered a fixed effect. Initial BW and sex were blocks which were considered random effects. The experimental unit was the pen for growth performance and fecal score data. The linear and quadratic effects of reducing ME levels and increasing xylanase levels were tested using polynomial contrasts. A preplanned contrast was executed using the CONTRAST statement to analyze the effects ME (ME 3,400 vs. others) and xylanase supplementation (0 vs. others). The individual pig representing the median BW of each pen from the selected treatments (CON, LE, and LEX) was the experimental unit for all additional measurements. A preplanned contrast was executed using the CONTRAST statement to analyze the effects of ME (CON vs. LE) and xylanase supplementation (LE vs. LEX). Results were considered statistically significant when *P* value was less than 0.05 and considered a tendency when *P* value was between 0.05 and 0.10.

## Results

### Digesta viscosity

**Figure 1.**
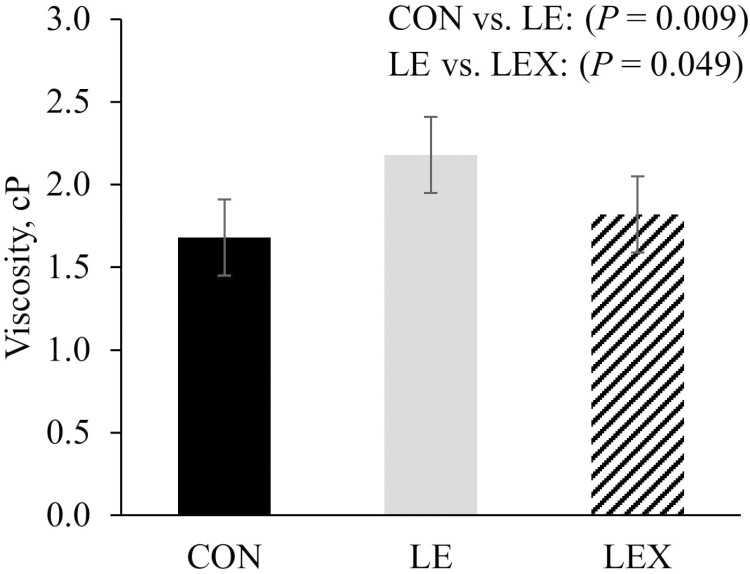
Changes in the viscosity of jejunal digesta in nursery pigs fed diets with reduced ME and xylanase supplementation. CON: metabolizable energy at 3,400 kcal ME/kg feed; LE: metabolizable energy at 3,300 kcal ME/kg feed; LEX: metabolizable energy at 3,300 kcal ME/kg feed and xylanase supplementation (3,600 XU/kg feed).

The viscosity of jejunal digesta was increased (*P* < 0.05) by LE, whereas was decreased (*P* < 0.05) by LEX (Figure 1).

### Diversity and relative abundance of jejunal mucosa-associated microbiota

At the family level, LE had no effect on Chao1 α-diversity, but tended to increase (*P* = 0.088) by LEX (Table 2). The LE tended to increase Simpson index at family (*P* = 0.058) and genus (*P* = 0.087) levels. The Shannon index was not affected by the treatments. Relative abundance of Bacteroidetes tended to be reduced (*P* = 0.071) by LE compared to CON, whereas it was increased (*P* < 0.05) by LEX (Table 3). Relative abundance of Proteobacteria was not affected by LE, whereas it was reduced (*P *< 0.05) by LEX. Relative abundance of *Prevotellaceae* tended to be reduced (*P* = 0.062) by LE compared to CON, whereas it was increased (*P* < 0.05) by LEX (Table 4). Relative abundance of *Helicobacteraceae* was not affected by LE, whereas it tended to be reduced (*P* = 0.072) by LEX. Relative abundance of *Prevotella* tended to be reduced (*P* = 0.053) by LE, whereas it was increased (*P* < 0.05) by LEX. Relative abundance of *Helicobacter* was unaffected by LE; however, tended to be reduced (*P* = 0.076) by LEX (Table 5). At the species level, LE had no effect on the relative abundance of *Prevotella copri*, whereas it was increased (*P* < 0.05) by LEX (Table 6). Interestingly, LE tended to increase the relative abundance of *Lactobacillus kitasatonis* (*P* = 0.073) and *Lactobacillus delbrueckii* (*P* = 0.054), whereas xylanase supplementation decreased (*P* < 0.05) the relative abundance of *L. delbrueckii.*

**Table 2.** α-Diversity of jejunal mucosa-associated microbiota at the family and genus level estimated with Chao1 richness, Shannon diversity, and Simpson diversity in nursery pigs fed diets with reduced ME and xylanase supplementation

Treatment^1^	CON	LE	LEX		*P* value	
ME/xylanase	3,400/0	3,300/0	3,300/3,600	SEM	CON vs. LE	LE vs. LEX
Family						
Chao1	6.54	7.02	10.70	1.81	0.823	0.088
Shannon	0.80	1.18	1.34	0.28	0.106	0.471
Simpson	0.37	0.58	0.58	0.13	0.058	1.000
Genus						
Chao1	6.00	5.57	7.86	1.61	0.794	0.142
Shannon	0.76	1.02	1.03	0.20	0.196	0.965
Simpson	0.35	0.54	0.48	0.11	0.087	0.540

^1^CON: metabolizable energy at 3,400 kcal ME/kg feed; LE: metabolizable energy at 3,300 kcal ME/kg feed; LEX: metabolizable energy at 3,300 kcal ME/kg feed and xylanase supplementation (3,600 XU/kg feed).

**Table 3.** Relative abundance of jejunal mucosa-associated microbiota at the phylum level in nursery pigs fed diets with reduced ME and xylanase supplementation

Treatment^1^	CON	LE	LEX		*P* value	
ME/xylanase	3,400/0	3,300/0	3,300/3,600	SEM	CON vs. LE	LE vs. LEX
Bacteroidetes	42.09	21.54	62.54	8.92	0.071	0.001
Proteobacteria	40.04	51.52	7.26	16.41	0.301	0.001
Firmicutes	16.32	25.52	24.14	7.81	0.258	0.839
Cyanobacteria	1.41	0.46	0.00	0.62	0.179	0.431
Deferribacteres	0.93	0.15	3.43	1.47	0.608	0.022
Lentisphaerae	0.00	0.00	0.07	0.03	1.000	0.112
Tenericutes	0.00	0.00	0.96	0.36	0.927	0.019

^1^CON: metabolizable energy at 3,400 kcal ME/kg feed; LE: metabolizable energy at 3,300 kcal ME/kg feed; LEX: metabolizable energy at 3,300 kcal ME/kg feed and xylanase supplementation (3,600 XU/kg feed).

**Table 4.** Relative abundance of jejunal mucosa-associated microbiota at the family level in nursery pigs fed diets with reduced ME and xylanase supplementation

Treatment^1^	CON	LE	LEX		*P* value	
ME/xylanase	3,400/0	3,300/0	3,300/3,600	SEM	CON vs. LE	LE vs. LEX
*Prevotellaceae*	42.03	20.22	61.56	9.51	0.062	0.001
*Helicobacteraceae*	29.83	31.89	5.21	14.70	0.900	0.072
*Lactobacillaceae*	7.01	19.11	9.40	3.97	0.086	0.104
*Veillonellaceae*	5.57	4.20	7.47	2.90	0.463	0.053
*Enterobacteriaceae*	4.31	5.48	0.27	4.49	0.796	0.190
*Lachnospiraceae*	2.58	0.12	2.55	1.05	0.136	0.089
*Succinivibrionaceae*	2.07	0.00	0.72	0.75	0.099	0.480
*Campylobacteraceae*	1.24	12.07	1.08	5.20	0.231	0.159
*Clostridiaceae*	0.94	1.37	1.02	0.85	0.652	0.666
*Deferribacteraceae*	0.93	0.15	3.43	1.47	0.609	0.022
*Brachyspiraceae*	0.84	0.92	0.33	0.54	0.926	0.410
*Streptococcaceae*	0.37	0.59	1.28	0.48	0.775	0.299
*Bacteroidaceae*	0.00	1.41	0.15	0.90	0.360	0.338
*Moraxellaceae*	0.00	1.84	0.43	1.16	0.257	0.351
Others	4.27	1.79	5.21	2.1	0.399	0.182

^1^CON: metabolizable energy at 3,400 kcal ME/kg feed; LE: metabolizable energy at 3,300 kcal ME/kg feed; LEX: metabolizable energy at 3,300 kcal ME/kg feed and xylanase supplementation (3,600 XU/kg feed).

**Table 5.** Relative abundance of jejunal mucosa-associated microbiota at the Genus level in nursery pigs fed diets with reduced ME and xylanase supplementation

Treatment^1^	CON	LE	LEX		*P* value	
ME/xylanase	3,400/0	3,300/0	3,300/3,600	SEM	CON vs. LE	LE vs. LEX
*Prevotella*	49.32	23.49	68.66	10.26	0.053	0.001
*Helicobacter*	33.67	34.38	6.75	14.89	0.967	0.076
*Lactobacillus*	9.19	26.98	10.66	5.09	0.055	0.041
*Succinivibrio*	2.32	0.00	0.86	1.13	0.102	0.447
*Campylobacter*	1.60	4.94	1.46	2.69	0.341	0.248
*Brachyspira*	1.05	0.38	0.50	0.72	0.324	0.830
*Clostridium*	0.82	1.96	1.25	1.29	0.395	0.534
*Megasphaera*	0.74	2.60	1.43	1.29	0.269	0.408
*Roseburia*	0.66	0.06	1.58	0.84	0.567	0.107
*Mucispirillum*	0.57	0.11	2.26	1.36	0.729	0.074
*Streptococcus*	0.53	0.74	1.44	0.73	0.478	0.235
*Acinetobacter*	0.00	2.78	0.56	2.22	0.270	0.336
*Pseudomonas*	0.00	1.47	0.21	1.26	0.358	0.359
Others	1.96	1.19	2.17	1.30	0.622	0.462

^1^CON: metabolizable energy at 3,400 kcal ME/kg feed; LE: metabolizable energy at 3,300 kcal ME/kg feed; LEX: metabolizable energy at 3,300 kcal ME/kg feed and xylanase supplementation (3,600 XU/kg feed).

**Table 6.** Relative abundance of jejunal mucosa-associated microbiota at the Species level in nursery pigs fed diets with reduced ME and xylanase supplementation

Treatment^1^	CON	LE	LEX		*P* value	
ME/xylanase	3,400/0	3,300/0	3,300/3,600	SEM	CON vs. LE	LE vs. LEX
*Prevotella copri*	52.46	28.26	65.61	11.21	0.164	0.028
*Helicobacter mastomyrinus*	19.69	23.70	4.28	8.12	0.770	0.138
*Prevotella stercorea*	5.66	5.37	12.42	3.35	0.960	0.187
*Helicobacter rappini*	13.06	7.72	1.37	6.21	0.096	0.267
*Lactobacillus kitasatonis*	2.87	11.30	1.75	2.87	0.073	0.029
*Campylobacterhyo intestinalis*	0.41	0.03	0.77	0.34	0.440	0.107
*Mucispirillum schaedleri*	0.00	0.17	3.32	1.68	0.940	0.136
*Roseburia faecis*	1.03	0.13	2.49	1.05	0.613	0.162
*Lactobacillus delbrueckii*	0.00	3.78	0.06	1.31	0.054	0.040
*Acinetobacter lwoffii*	0.00	2.90	0.00	1.75	0.220	0.205
*Brachyspira hampsonii*	1.30	0.96	0.59	1.09	0.744	0.697
*Lactobacillus mucosae*	0.18	1.57	0.22	0.79	0.139	0.112
Others	1.96	1.19	2.17	1.30	0.622	0.462

^1^CON: metabolizable energy at 3,400 kcal ME/kg feed; LE: metabolizable energy at 3,300 kcal ME/kg feed; LEX: metabolizable energy at 3,300 kcal ME/kg feed and xylanase supplementation (3,600 XU/kg feed).

### Intestinal inflammatory status, humoral immune status, and oxidative stress status

The concentrations of pro-inflammatory cytokines (IL-8 and TNF-a) and immunoglobulins (IgA and IgG) in the jejunal mucosa were not affected by LE or LEX (Table 7). Protein carbonyl concentration in the jejunal mucosa was increased (*P* < 0.05) by LE, whereas it was reduced (*P *< 0.05) by LEX. Malondialdehyde (MDA) concentration in the jejunal mucosa was reduced (*P* < 0.05) by LE, whereas it was not influenced by LEX.

**Table 7.** Pro-inflammatory cytokines, oxidative damage products, and immunoglobulins in the jejunal mucosa (mg protein) of pigs fed diets with reduced ME and xylanase supplementation

Treatment^1^	CON	LE	LEX		*P* value	
ME/xylanase	3,400/0	3,300/0	3,300/3,600	SEM	CON vs. LE	LE vs. LEX
Unit/mg protein						
IL-8^2^, ng	0.80	0.62	0.65	0.08	0.137	0.792
TNF-α^3^, pg	1.17	1.20	0.95	0.33	0.942	0.535
PC^4^, nmol	0.18	0.40	0.17	0.04	0.033	0.049
MDA^5^, nmol	0.70	0.39	0.40	0.10	0.036	0.928
IgG^6^, µg	2.41	2.74	2.74	0.52	0.537	0.993
IgA^7^, µg	6.50	7.72	7.08	0.35	0.572	0.766

^1^CON: metabolizable energy at 3,400 kcal ME/kg feed; LE: metabolizable energy at 3,300 kcal ME/kg feed; LEX: metabolizable energy at 3,300 kcal ME/kg feed and xylanase supplementation (3,600 XU/kg feed).

^2^Interlukin-8.

^3^Tumor necrosis factor α.

^4^Protein carbonyl.

^5^Malondialdehyde.

^6^Immunoglobulin A.

^7^Immunoglobulin G.

### Intestinal morphology and crypt cell proliferation

VH was reduced (*P *< 0.05) by LE, whereas it tended to be increased (*P* = 0.065) by LEX (Table 8). Crypt depth (CD) was not influenced by LE, whereas it was reduced (*P* < 0.05) by LEX. **VH:CD** was reduced (*P *< 0.05) by LE, whereas it increased (*P* < 0.05) by LEX. Percentage of proliferating crypt cells was not influenced by LE and LEX. However, the number of proliferating cells in a crypt was reduced (*P* < 0.05) by LEX.

**Table 8.** Morphology of jejunum in nursery pigs fed diets with reduced ME and xylanase supplementation

Treatment^1^	CON	LE	LEX		*P* value	
ME/xylanase	3,400/0	3,300/0	3,300/3,600	SEM	CON vs. LE	LE vs. LEX
Villus height, µm	467	433	462	14.73	0.032	0.065
Crypt depth, µm	264	263	238	8.47	0.916	0.039
VH:CD^2^	1.80	1.64	1.99	0.12	0.041	0.022
Ki-67^+^^3^, %	26.1	27.5	24.3	0.02	0.548	0.160
Ki-67^+^^4^, count	78.3	82.8	69.9	3.1	0.324	0.009

^1^CON: metabolizable energy at 3,400 kcal ME/kg feed; LE: metabolizable energy at 3,300 kcal ME/kg feed; LEX: metabolizable energy at 3,300 kcal ME/kg feed and xylanase supplementation (3,600 XU/kg feed).

^2^Villus height to crypt depth ratio.

^3^Ratio of Ki-67 positive cells to total cells in the crypt.

^4^Number of Ki-67 positive cells in the crypt.

### Apparent ileal digestibility of nutrients

Apparent ileal digestibility of DM, GE, CP, and EE was reduced (*P* < 0.05) by LE, whereas digestibility of DM and GE was increased (*P* < 0.05) by LEX and digestibility of CP (*P* = 0.099), EE (*P* = 0.076), and crude fiber (*P* = 0.060) tended to be increased by LEX (Table 9).

**Table 9.** AID of nutrients in nursery pigs fed diets with reduced ME and xylanase supplementation

Treatment^1^	CON	LE	LEX		*P* value	
ME/xylanase	3,400/0	3,300/0	3,300/3,600	SEM	CON vs. LE	LE vs. LEX
AID^2^ of nutrients, %						
Dry matter	59.52	47.28	58.66	3.80	0.008	0.019
Gross energy	59.14	42.98	56.00	3.48	0.001	0.001
Crude protein	63.40	44.63	57.15	5.13	0.012	0.099
Crude fiber	67.73	62.44	69.10	3.31	0.102	0.060
Ether extract	67.73	55.22	64.39	4.84	0.013	0.076

^1^CON: metabolizable energy at 3,400 kcal ME/kg feed; LE: metabolizable energy at 3,300 kcal ME/kg feed; LEX: metabolizable energy at 3,300 kcal ME/kg feed and xylanase supplementation (3,600 XU/kg feed).

^2^Apparent ileal digestibility.

### Growth performance and fecal score

BW, ADG, and G:F were not affected during any of the experimental periods by reducing energy up to 100 kcal ME/kg of feed or the supplementation of xylanase to LE (Table 10). Pigs fed the CON treatment had increased (*P* < 0.05) ADFI compared to the 3,375 ME kcal/kg, 3,350 ME kcal/kg, and LE (3,300 ME kcal/kg) treatments during the pre-starter phase and tended to have higher (*P* = 0.064) ADFI for the overall period compared to 3,375 ME kcal/kg and 3,350 ME kcal/kg treatments. Reducing energy up to 100 kcal ME/kg of feed had no effect on fecal score throughout the trial, however, increasing xylanase supplementation to LE tended to decrease (*P* = 0.070) the fecal score during starter phase (Table 11).

**Table 10.** Effects of energy contents on growth performance in nursery pigs

Treatment	ME^1^, kcal/kg					Xylanase^2^, XU/kg				*P* value					
Item	3,400	3,375	3,350	3,325	3,300	1,200	2,400	3,600	SEM	ME linear^3^	ME quad^3^	ME 3,400 vs. others^3^	Xylanase linear^4^	Xylanase quad^4^	LE vs. xylanase^4^
BW, kg															
Initial	7.3	7.3	7.3	7.3	7.3	7.3	7.3	7.3	0.6	0.972	0.980	0.991	0.982	0.990	0.980
Day 7	8.8	8.6	8.9	8.6	8.7	8.6	8.4	8.8	0.7	0.853	0.900	0.658	0.906	0.173	0.577
Day 14	10.9	10.5	11.1	10.6	10.5	10.5	10.7	10.7	1.1	0.471	0.708	0.440	0.620	0.949	0.820
Day 21	14.0	12.6	13.7	13.3	13.8	12.9	13.3	13.6	1.2	0.833	0.207	0.225	0.953	0.233	0.358
Day 28	18.7	17.2	18.4	17.9	17.7	17.1	17.6	18.0	1.5	0.431	0.664	0.149	0.566	0.397	0.859
Day 35	23.0	21.4	22.5	21.8	22.3	21.7	21.9	22.2	2.0	0.639	0.407	0.232	0.980	0.520	0.686
ADG, g															
Pre-starter	260	227	275	237	232	227	244	241	35	0.368	0.633	0.326	0.533	0.950	0.775
Starter	574	522	540	532	558	533	531	550	45	0.779	0.175	0.204	0.823	0.387	0.494
Overall	449	404	434	414	427	410	417	426	40	0.609	0.368	0.192	0.975	0.496	0.670
ADFI, g															
Pre-starter	600^a^	535^b^	590^a^	547^b^	552^b^	547	560	560	37	0.116	0.500	0.025	0.642	0.895	0.858
Starter	860	744	837	807	812	762	805	823	78	0.759	0.368	0.127	0.615	0.329	0.707
Overall	838^a^	746^b^	808^ab^	774^ab^	783^ab^	755	778	788	67	0.365	0.327	0.064	0.765	0.500	0.774
G/F															
Pre-starter	0.45	0.42	0.46	0.42	0.42	0.41	0.43	0.43	0.04	0.462	0.623	0.571	0.631	0.772	0.990
Starter	0.67	0.70	0.65	0.66	0.69	0.70	0.66	0.67	0.02	0.977	0.359	0.772	0.265	0.984	0.612
Overall	0.54	0.54	0.54	0.53	0.55	0.54	0.53	0.54	0.01	0.691	0.599	0.812	0.651	0.582	0.582

^1^Metabolizable energy level at 3,400 kcal/kg of feed (CON), 3,375 kcal/kg of feed, 3,350 kcal/kg of feed, 3,325 kcal/kg of feed, and 3,300 (LE) kcal/kg of feed.

^2^Xylanase supplemented at 0 (LE), 1,200, 2,400, and 3,600 XU/kg feed (LEX) to LE treatment (3,300 kcal/kg feed).

^3^Dose–response effects of ME level (3,400 kcal/kg of feed [CON], 3,375 kcal/kg of feed, 3,350 kcal/kg of feed. 3,325 kcal/kg of feed, and 3,300 kcal/kg of feed [LE]).

^4^Dose response effects of xylanase supplementation (xylanase at 0 XU/kg feed [LE], 1,200 XU/kg feed, 2,400 XU/kg feed, and 3,600 XU/kg feed [LEX]).

**Table 11.** Fecal score of nursery pigs fed diets with reduced ME and xylanase supplementation

Treatment	ME^1^, kcal/kg					Xylanase^2^, XU/kg				*P* value			
Item	3,400	3,375	3,350	3,325	3,300	1,200	2,400	3,600	SEM	ME Linear^3^	ME Quad.^3^	Xylanase Linear^4^	Xylanase Quad.^4^
Pre-starter	3.3	3.1	3.2	3.3	3.1	3.2	3.2	3.2	0.1	0.799	0.830	0.529	0.840
Starter	3.3	3.2	3.3	3.3	3.4	3.0	3.3	3.1	0.1	0.250	0.515	0.070	0.288
Overall	3.3	3.2	3.3	3.3	3.3	3.1	3.3	3.1	0.1	0.503	0.572	0.350	0.526

^1^Metabolizable energy level at 3,400 kcal/kg of feed (CON), 3,375 kcal/kg of feed, 3,350 kcal/kg of feed, 3,325 kcal/kg of feed, and 3,300 (LE) kcal/kg of feed.

^2^Xylanase supplemented at 0 (LE), 1,200, 2,400, and 3,600 XU/kg feed (LEX) to LE treatment (3,300 kcal/kg feed).

^3^Dose–response effects of ME level (3,400 kcal/kg of feed (CON), 3,375 kcal/kg of feed, 3,350 kcal/kg of feed. 3,325 kcal/kg of feed, and 3,300 kcal/kg of feed (LE)).

^4^Dose–response effects of xylanase supplementation (xylanase at 0 XU/kg feed [LE], 1,200 XU/kg feed, 2,400 XU/kg feed, and 3,600 XU/kg feed [LEX]).

## Discussion

Pigs fed LE had increased viscosity of jejunal digesta whereas, LEX decreased digesta viscosity compared to LE. Alterations to the viscosity of the digesta changes the intestinal environment and can modulate the exacerbation or onset of infectious intestinal diseases (Bertschinger et al., 1979; McDonald et al., 1999). Increased digesta viscosity in nursery pigs has been shown reduce starch digestibility and worsen postweaning diarrhea (Hopwood et al., 2004) and has been shown to increase the rate of enterocyte apoptosis leading to villus atrophy ([Bibr CIT0062][Bibr CIT0029]).

Xylanase supplementation seemed to increase Chao1 alpha diversity of mucosa-associated microbiota in the jejunum and this could be related to enhanced maintenance and stabilization of the ecosystem allowing it to retain its resilience against potential pathogenic invasion and proliferation (Konopka, 2009). Moreover, a more diverse microbiota has been regarded as a sign of a mature intestine environment in pigs (Chen et al., 2017). The changes in the alpha diversity observed in the current study resulted from distinct alterations in the composition of the microbiota in the jejunal mucosa.

Firmicutes are the predominant phylum in the lumen of the small intestine in nursery pigs whereas Bacteroidetes are the predominant phylum in the feces of postweaning pigs (Pajarillo et al., 2014; Adhikari et al., 2019). Age-dependent alterations in microbial communities in pigs have been shown by an increase in the relative abundance of Bacteroidetes in the jejunal mucosa and feces as nursery pigs age whereas Firmicutes reduced (Bian et al., 2016; Adhikari et al., 2019). Importantly, the population of microbiota in feces largely differs compared with those in the jejunal mucosa (Adhikari et al., 2019; Duarte and Kim, 2022). The relative abundance of both Proteobacteria and Bacteroidetes have been shown to be higher in mucosa compared to the lumen (Chen et al., 2017; Mu et al., 2017; Adhikari et al., 2019) which would explain the high abundance seen in the present study as samples were obtained from jejunal mucosa. Characterizing the mucosa-associated microbiota may be more relevant when evaluating their roles in the intestinal immune responses of the host (Mulder et al., 2011; Daniel et al., 2021). According to Arpaia et al. (2013) and Belkaid and Hand (2014), the microbiota associated with the mucosa has been shown to directly interact with intestinal cells. Moreover, the mucosa-associated microbiota was reported to have a greater ability to modulate the host’s immune system compared with luminal microbiota (Mu et al., 2017). Furthermore, Liu et al. (2019) reported that characterizing the fecal microbiota may be inefficient for comprehending the interactions between the intestinal microbiota and the host’s immune system.

The LEX lowered the relative abundance of Proteobacteria in the jejunal mucosa. This could have important implications as Proteobacteria contain several Gram-negative pathogenic bacteria such as *Escherichia*, *Salmonella*, *Helicobacter*, *Campylobacter*, and *Vibrio* that are used as potential indicators of intestinal dysbiosis (Shin et al., 2015). Gram-negative bacteria are characterized by possessing an outer cell wall with lipopolysaccharides (LPS) that provide integrity to the bacterial cell and act as a mechanism of interaction of the bacteria to outer surfaces (Li et al., 2017). Most bacterial LPS are thermostable and able to elicit a strong pro-inflammatory stimulus to the immune system of mammals (Sun and Kim, 2017). Indeed, xylanase reduced the relative abundance of *Helicobacter* in the jejunal mucosa which may indicate a healthier intestinal microbiota as previous reported by Cheng et al. (2021) and Xu et al. (2022). The most abundant genus in the jejunal mucosa in this study was *Prevotella* in the jejunal mucosa of nursery pigs fed an ME sufficient diet (CON) and an ME deficient diet with xylanase (LEX). Fecal *Prevotella* spp. has been shown to have positive associations with feed intake, feed efficiency, and weight gain (Mach et al; 2015; Ramayo-Caldas et al., 2016; Yang et al., 2018). However, in the present study, the enrichment of *Prevotella* in jejunal mucosa was not related to growth improvement. Species belonging to *Prevotella* have abilities to breakdown complex plant cell walls by utilizing endogenously produced NSPases such as xylanase, mannanase, and β-glucanases (Flint et al., 2008).

Interestingly, the relative abundance of *Lactobacillus* was high in the jejunal mucosa of nursery pigs fed an ME deficient diet (LE) compared to CON and LEX treatments. Several studies have indicated that *Lactobacillus* species improve growth and decrease the incidence of diarrhea (Dowarah et al., 2017; Yi et al., 2018; Wang et al., 2019). In the present study, the increase in *Lactobacillus* observed in LE was not related to reduced intestinal inflammation, reduced oxidative stress, enhanced intestinal integrity, and improved growth indicating the need for more research into how different microbial communities work together to produce a specific phenotype rather than increases or decreases in one particular genus. Additionally, the relative abundance of *Mucispirillium* was increased in the jejunal mucosa of nursery pigs fed an ME deficient diet with xylanase (LEX), mostly due to the increase in *Mucispirillum schaedler,* though no effect was observed at the species level. *Mucispirillum schaedler* has been shown to have a very limited collection of glycoside hydrolases, with only 3 family 57 α-amylases that are thought to be primarily used for processing stored glycogen (Loy et al., 2017). Therefore, *M. schaedler* is not a primary degrader of host-derived glycans in mucin but rather uses monosaccharides, oligopeptides, amino acids, and SCFAs as the substrates for its energy metabolism and is likely a consumer of products formed by other fermentative bacteria (El Kaoutari et al., 2013).

Feeding nursery pigs with an ME deficient diet of xylanase (LEX) did not affect the concentration of pro-inflammatory cytokines (IL-8 and TNF-α) or immunoglobulins (IgG and IgA) whereas, pigs fed an ME deficient diet (LE) had higher concentrations of protein carbonyl and lower amounts of MDA compared to pigs fed an ME sufficient diet. Xylanase supplementation to an ME deficient diet decreased concentrations of protein carbonyl. Weaning stress has been shown to induce oxidative stress in weaned pigs (Yin et al., 2014) and oxidative damage products can be detrimental to the intestinal epithelium, causing cell destruction and resulting in a reduction of villi height (Sido et al., 2017). The results in the present study support this as xylanase supplementation reduced the concentration of protein carbonyl in the mucosa compared with increased VH and VH:CD ratio. Reduction in oxidative damage products could be due to increased bioavailability of phenolic compounds with antioxidative properties. These phenolic compounds can be generated during the hydrolysis of arabinoxylan structure within the fiber fraction of corn (Duarte et al., 2019; Petry et al, 2020). Arabinoxylan in corn is highly substituted with phenolic compounds with the most abundant being ferulic acid (Boz, 2015). Ferulic acid has been shown to possess strong antioxidant abilities through the scavenging of free radicals, inhibition of enzymes that cause free radical production and stimulating antioxidase production (Ogiwara et al., 2002). In typical diets devoid of xylanase supplementation, the bioavailability of ferulic acid is limited due to esterification within arabinoxylan (Anson et al., 2009), however, supplementation of xylanase may fragment the arabinoxylan structure possibly allowing the access of esterified ferulic acid to ferulic acid esterase produced by microbiota (Mathew and Abraham, 2004).

Supplementation of xylanase has been shown to increase nutrient digestibility and thus growth performance of pigs (Patience, 2012). In this study, pigs fed an ME deficient diet with xylanase had increased AID of DM and GE and tended to have increased AID of CP, EE, and crude fiber, whereas this did not translate to improved growth. This result is in agreement with a meta-analysis published by Torres-Pitarch et al. (2019) that found regardless of diet composition, DM, GE, and CP digestibility increased with xylanase supplementation but resulting enhancements in growth performance were not as frequently observed. This is not uncommon when evaluating the efficacy of xylanase to improve growth as improvements in digestibility and subsequent responses in growth performance are inconsistent in corn-based diets and time-dependent (Kerr et al., 2013; Petry et al., 2020). Inconsistency in growth responses to xylanase among studies most likely can be attributed to inadequate determination of fiber within the feed offered, differences in age, breed, feeding duration, and feedstuffs used in conjunction with corn thus resulting in alterations to fermentation kinetics, digesta viscosity, and the microbiota composition. Additionally, the inclusion rate of xylanase in the diet, the presence or absence of supporting enzymes or xylanase inhibitors, and health status of the animal may play a role in the observation of a growth response when utilizing xylanase in formulation.

In the present study, reducing ME (LE) had no effect on overall BW, ADG, ADFI, or G:F. Although increasing energy density of diets offered to pigs through the inclusion of dietary fat typically improves ADG, G:F, and reduces ADFI (Pettigrew and Moser, 1991), this may not be completely appropriate when discussing pigs weighing less than 20 kg (Black et al., 1986) as they possess immature and limited digestive capacity which ultimately restricts their ability to adjust their feed intake relative to adult animals.

In conclusion, reducing ME up to 100 kcal/kg below the requirement by reducing supplemental fat had no effect on overall growth performance, however, it led to increased digesta viscosity, decreased diversity of mucosa-associated microbiota, increased relative abundance of potentially pathogenic bacteria in the jejunal mucosa, and elevated the concentration of oxidative damage products in the jejunal mucosa. These negative effects were seen by reducing ME manifested in negative effects on VH and VH:CD in jejunum and decreased nutrient digestibility. Supplementation of xylanase to an ME deficient diet helped to mediate some of the negative effects induced by energy restriction, resulting in increased diversity of mucosa-associated microbiota and the relative abundance of fiber-degrading bacteria, decreased oxidative damage products, reduced digesta viscosity, increased VH, and improved nutrient digestibility which are indicative of improvements in intestinal health that may be beneficial in subsequent phases of production.

## Abbreviations

ADFI: average daily feed intake
ADG: average daily gain
AID: apparent ileal digestibility
AX: arabinoxylan
BW: body weight
CD: crypt depth
CP: crude protein
cP: centipoise
DM: dry matter
GE: gross energy
G:F: gain to feed ratio
EE: ether extract
IgA: immunoglobulin A
IgG: immunoglobulin G
IL-8: interleukin-8
IL-6: interleukin-6
LPS: lipopolysaccharide
MDA: malondialdehyde
ME: metabolizable energy
mPA s: millipascal-seconds
NSP: nonstarch polysaccharide
PBS: phosphate-buffered saline
PC: protein carbonyl
PUFA: polyunsaturated fatty acid
SCFA: short chain fatty acid
SID: standardized ileal digestible
TNF-α: tumor necrosis factor alpha
VH: villus height
